# Surgical Incision Induces Anxiety-Like Behavior and Amygdala Sensitization: Effects of Morphine and Gabapentin

**DOI:** 10.1155/2010/705874

**Published:** 2010-03-04

**Authors:** Chang-Qi Li, Jian-Wei Zhang, Ru-Ping Dai, Juan Wang, Xue-Gang Luo, Xin-Fu Zhou

**Affiliations:** ^1^Department of Anesthesia, The Second Xiang-Ya Hospital of Central South University, Renmin Road no.86, Changsha, Hunan 410011, China; ^2^Department of Anatomy and Neurobiology, Xiang-Ya College of Medicine, Central South University, Tongzipo Road 168, Changsha, Hunan 410089, China; ^3^Department of Human Physiology and Center for Neuroscience, Flinders University, GPO Box 2100, Adelaide SA5001, Australia

## Abstract

The role of affective dimension in the postoperative pain is still poorly understood. The present study investigated the development of anxiety-like behavior and amygdala sensitization in incisional pain. Using hind-paw incision model in rats, we showed that surgical incision induced the anxiety-like behavior as determined by elevated plus-maze and open-field tests. Intraperitoneal (IP) morphine administration reversed mechanical allodynia and anxiety-like behavior in a dose-dependent manner. Gabapentin also partially reduced incision-evoked mechanical allodynia and anxiety-like behavior in a dose-dependent manner. After incision, the expression of phosphorylated cAMP response elements (CRE-) binding protein (p-CREB) was transiently upregulated in the central and basolateral nuclei in the bilateral amygdala. The upregulation of p-CREB was inhibited by morphine and gabapentin. The present study suggested that surgical incision could induce anxiety and amygdala sensitization that can be inhibited by morphine and gabapentin. Thus treatment of surgery-induced affective disturbances by morphine and gabapentin may be a potential important adjunct therapy in the postoperative pain management.

## 1. Introduction

Pain can be thought of as having sensory dimension and affective dimension which is made up of feelings of unpleasantness and emotions [1]. Numerous studies have shown that various chronic pain states are accompanied by the negative affects such as anxiety and depression [2–5]. The affective disturbances have a reciprocal relationship with chronic pain in which each disorder may make the patients more vulnerable to the other disorder. For example, patients with chronic pain easily developed depression whereas anxiety could promote the chronic pain state [6]. On the other hand, experimental animal studies suggest that pain reactivity is decreased by fear [7, 8] whereas anxiety decreased pain threshold [9, 10]. The accompanied negative affects in patients with chronic pain are very important comorbidities and decrease life quality [2, 11, 12]. However, most studies about the roles of affects and its relationship with pain sensation are limited in the chronic pain states [13–15]. It still remains to be determined the role of affective dimension in postoperative pain, an acute pain state.

 Pain induced by surgical injury is more transitory and still a challenge for the postoperative management. In spite of the fact that the optimal management of postoperative pain also includes the alleviation of perioperative anxiety [16], the role of anxiety in postoperative pain has not been studied, in particular in animal models. Using a hind-paw incision model in rats, the present study investigated the development of anxiety-like behavior and the effect of analgesics, morphine and gabapentin, on the pain-related anxiety in incisional pain. The present study may shed some light on the role of anxiety in the postoperative pain.

## 2. Materials and Methods

### 2.1. Animals

The study was carried out on male Wistar rats (150–250 g) obtained from Central South University Animal Services (Changsha, China). The experimental protocol was approved by the Animal Care and Use Committee of Central South University and conformed to the National Institutes of Health Guide for the Care and Use of Laboratory Animals. All efforts were made to minimize the number of rats used and their suffering. 

Gabapentin was a gift from En-Hua Pharmaceutical Company (Xu-Zhou, China) and morphine was obtained from Shen-Yang First Pharmaceutical Company (Shen-Yang, China). All compounds are dissolved in saline and were administered intraperitoneally (IP).

### 2.2. Surgical Preparation

Animals were briefly anesthetized with 1.5% isoflurane in oxygen, and an incision was made in one hind paw as described previously [17]. Using sterile technique, a 1 cm long longitudinal incision was made into the right hind plantar skin with a number 11 scalpel blade, starting 0.5 cm from the edge of the heel. The plantaris muscle was elevated and incised longitudinally (0.5 cm) with the blade. The skin was closed with 4-0 nylon sutures, and a topical triple antibiotic ointment was applied to the plantar hind paw. The sham-operated animals underwent the same procedure except that the incision was not carried out.

### 2.3. Elevated Plus-Maze (EPM) Test

Anxiety-like behavior was tested by EPM test as described previously [14]. The EPM test was performed in an apparatus comprising two open arms and two closed arms (45 × 40 × 10 cm) that extended from a common central platform (10 × 10 cm). The maze was constructed from black materials and kept to a height of 80 cm above floor level. After adaptation around one day in the experimental room, rats were placed in the middle of the EPM and the behavior was recorded by a video camera for 10 minutes. The percentage of time spent in open arms was recorded and used for statistical analysis.

### 2.4. Open-Field Test

Animals were removed from the home cage and placed directly into one corner of the open field (120 cm×120 cm) [18]. The floor was divided into a grid of 8  ×  8 squares. Movement of the animal in the arena during the 10-minute testing session was recorded. After 10 minutes, the animal was removed and returned to the home cage, and the open-field arena was cleaned to prevent olfactory cues from affecting the behavior of subsequently tested rats. An observer blind to the experimental conditions coded the videotapes. Exploration was defined as the time spent in the inner 6 × 6 squares, whereas overall activity was defined as the number of squares crossed during the testing session.

### 2.5. Nociceptive Testing

Mechanical allodynia was assayed using nylon von Frey filaments according to the “up-down” algorithm described by Chaplan et al. [19]. The nociceptive testing was performed by two authors who were blind to the experimental treatment. In these experiments, rats were placed on wire mesh platforms in clear cylindrical plastic enclosures of 10 cm diameter. After 20 minutes of acclimation, fibers of sequentially increasing stiffness were applied to the center of the plantar surface of the right hind paw between the first set of foot pads and left in place 5 s. For incisional animals, the fibers were placed directly on the wound edge. Withdrawal of the hind paw from the fiber was scored as a response. When no response was obtained, the next stiffer fiber in the series was applied to the same paw; if a response was obtained, a less stiff fiber was next applied. Testing proceeded in this manner until four fibers had been applied after the first one causing a withdrawal response allowing the estimation of the mechanical withdrawal threshold.

### 2.6. Immunohistochemical Protocol

The brains from the control and experimental rats were fixed for 4 hours with 2% paraformaldehyde after perfusion and cryoprotected by immersion in 20% sucrose in phosphate buffer (pH 7.4) overnight. Transverse sections of the brain were cut at cryostat and mounted on 3-aminopropyl triethoxy-silane-coated slides; mouse anti-p-CREB antibody (dilution 1 : 200; Chemicon, USA) was incubated at room temperature overnight. The secondary reagents used for localization were biotinylated goat anti-mouse IgG and ABC kit (Vector Laboratories, USA). The -diaminobenzidine tetrahydrochloride (DAB, Sigma, USA) was used as a peroxidase substrate. In order to define the density of p-CREB-IR, relative optic density (OD) of positive staining was performed using HPIAS-1000 image analysis as described in our recent studies [20]. The measurements were performed by an author who was blind with respect to treatments.

## 3. Experimental Design

### 3.1. Experiment I

The first aim was to investigate whether animals with surgical pain develop anxiety-like behavior. EPM test was carried out in six groups of rats with baseline (BL) and at different times (1 hour, 3 hours, 6 hours, 24 hours, 72 hours, and 7 days) after surgical incision. Animals for EPM test were tested only one time at the indicated time. Open-field test in an independent group was also performed at 1 hour after surgery to further confirm the anxiety-like behavior. In a third independent group, paw-withdrawal threshold (PWT) was measured after surgical incision to confirm to confirm the findings as reported previously [21].

### 3.2. Experiment II

To explore the effect of analgesic drugs on anxiety-like behavior and pain sensation, rats with hind-paw incision were treated with saline, two doses of morphine (2.5 mg/kg and 10 mg/kg, respectively), two doses of gabapentin (30 mg/kg and 300 mg/kg, respectively), or combined morphine (2.5 mg/kg) and gabapentin (30 mg/kg). The EPM and PWT tests were performed to investigate the effect of those compounds on anxiety-like behavior and mechanical hypersensitivity.

### 3.3. Experiment III

 In an independent group, p-CREB expression in the amygdala was examined to investigate the involvement of amygdala sensitization in the anxiety-like behavior. Rats in various experimental groups in experiment I and II were sacrificed at the indicated times immediately after nociceptive testing and the brain were used for immunohistochemistry.

### 3.4. Statistical Analysis

Statistical analysis was performed using software SPSS 13.0 (SPSS Inc.). Data are given as mean ± SEM. The assessment of pain and anxiety-like behavior after incision was accomplished using one-way analysis of variance (ANOVA) followed by post hoc Dunnett testing. The experiments to investigate the effect of drugs on pain and anxiety were analyzed by performing a parametric two-way ANOVA test followed by Bonferroni testing. A value of *P* < .05 was considered as significant difference.

## 4. Results

### 4.1. Surgical Incision Induces Mechanical Hypersensitivity and Anxiety-Like Behavior

**Figure 1:**
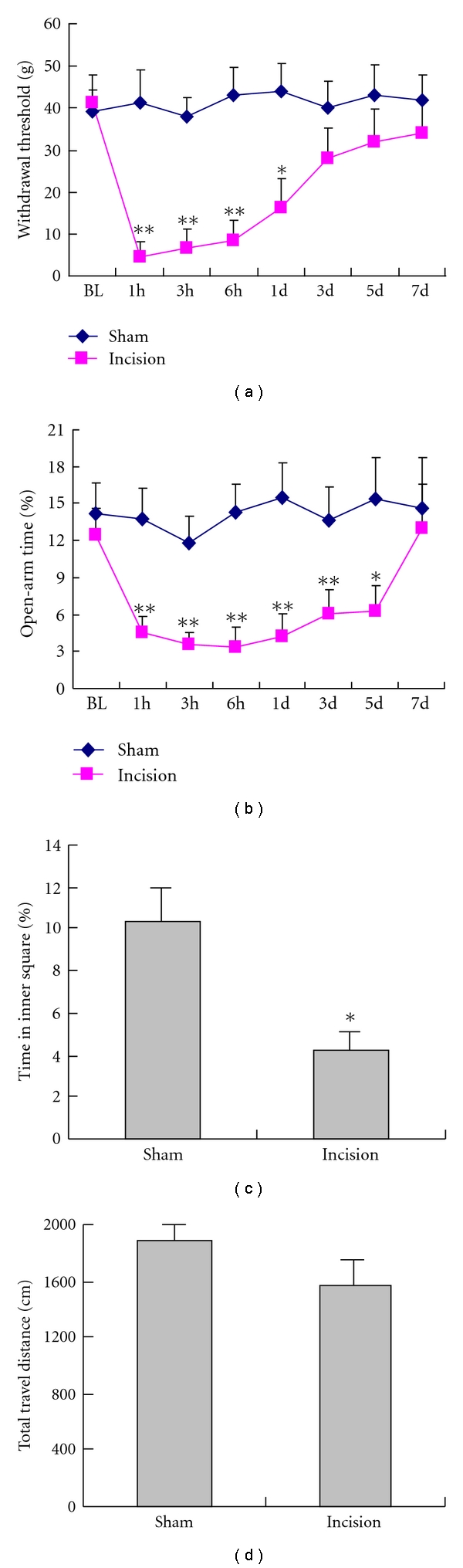
Development of an anxiety-like behavior and mechanical allodynia following surgical incision. (a) Time-course changes in PWT in the rats with surgical incision and (b) the percentage of time spent in open arm in the EPM tests in the sham-operated and incisional groups. (c) Open-field test shows the decreased time spent in the inner square at 1 hour after operation. (d) Total travel distance in open-field test between the control and 1 hour postoperative rats. *P < .05, **P < .01 were compared to the sham-operated control (*n* = 7). BL indicates baseline.

The paw-withdrawal threshold (PWT) in the operated paw dramatically decreased from 43.3 ± 5.2 g to 4.4 ± 2.2 g as early as 1 hour after right hind-paw surgical incision (Figure 1(a); *P* < .01). The decreased PWT sustained more than 1 day after operation as compared with the sham operation (*P* < .05). At 3 days after surgery, the PWT was returned to basal level and was comparable to the sham operation (Figure 1(a)).

After hind-paw incision, the time spent in open arm in EPM tests was greatly reduced as compared with the basal level or sham operation, suggesting that incision leads to anxiety-like behavior (*P* < .05, Figure 1(b)). Interestingly, the pain-related anxiety remained up to five postoperative days when the time spent in open arm in the experimental group was still significantly lower than that in the sham operated groups (*P* < .05, Figure 1(b)). 

 Open-field test showed that the percentage of time spent in the inner square was significantly decreased from 11 ± 1.5% in the sham operation to 3.8 ± 1.2% at 1 hour after surgery (*P* < .05, Figure 1(c)). However, there was no significant difference in the total travel distance between the control and the experimental groups (*P* > .05, Figure 1(d)). These results indicated that incision-evoked anxiety-like behavior was not due to the impaired locomotion induced by surgical trauma.

### 4.2. Transient Activation of p-CREB in the Amygdala in Response to Hind-Paw Incision

**Figure 2:**
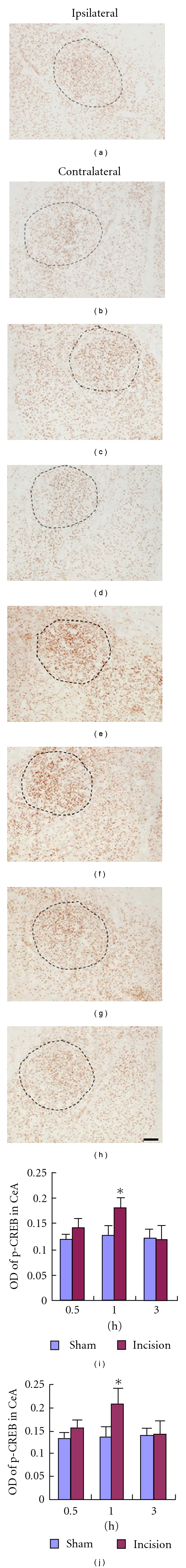
Effect of hind-paw incision on the p-CREB expression in the CeA of amygdala. Expression of p-CREB in the bilateral CeA of amygdala at 1 hour after sham-operation (a) and (b), at 30 minutes (c) and (d), 1 hour (e) and (f), as well as 3 hours (g) and (h) after surgical incision. The CeA in amygdala are displayed by the labeled circle; (i) and (k) show the quantitative analysis of the OD of p-CREB in the bilateral CeA at indicated times after incision. Bar, 300 m. *P < .05 versus the sham-operated control (*n* = 5 for each group per indicated time).

As shown in Figure 2, constitutive mild staining of p-CREB immunoreactivity was observed in the bilateral CeA of the amygdala (Figures 2(a) and 2(b)). At 0.5 hour after incision, the expression of p-CREB in the bilateral CeA was still comparable with the sham operation (Figures 2(c) and 2(d)). However, at 1 hour after operation, the p-CREB expression was significantly increased in the ipsilateral (Figure 2(e)) and contralateral (Figure 2(f)) sides of CeA (*P* < .05 versus sham operation). At 3 hours after incision, p-CREB expression was declined and was comparable with the sham operation (*P* > .05). 

**Figure 3:**
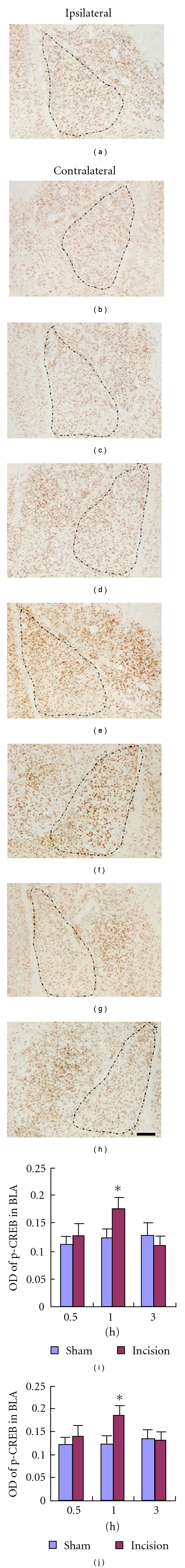
Effect of incision on p-CREB expression in the BLA of amygdala. Expression p-CREB in the bilateral BLA in the sham operation (a) and (b), at 30 minutes (c) and (d), 1 hour (e) and (f) and 3 hours (g) and (h) after hind-paw incision. (i) and (k) Quantitative analysis of the OD of p-CREB in bilateral BLA after hind-paw incision. Bar, 300 m. *P < .05 versus the sham operation (*n* = 5 for each group).

Similarly, p-CREB was mildly expressed in bilateral BLA of amygdala in the sham-operation (Figures 3(a) and 3(b)) and at 30 minutes after surgical incision. In response to surgical incision, the staining of p-CREB immunoreactivity was significantly intensified in the bilateral BLA at 1 hour after incision (Figures 3(e) and 3(f); *P* < .05). The increased p-CREB expression returned to the basal level at 3 hours after incision.

### 4.3. Effect of Morphine and Gabapentin on Mechanical Hypersensitivity and Anxiety-Like Behavior in Incisional Rats

**Figure 4:**
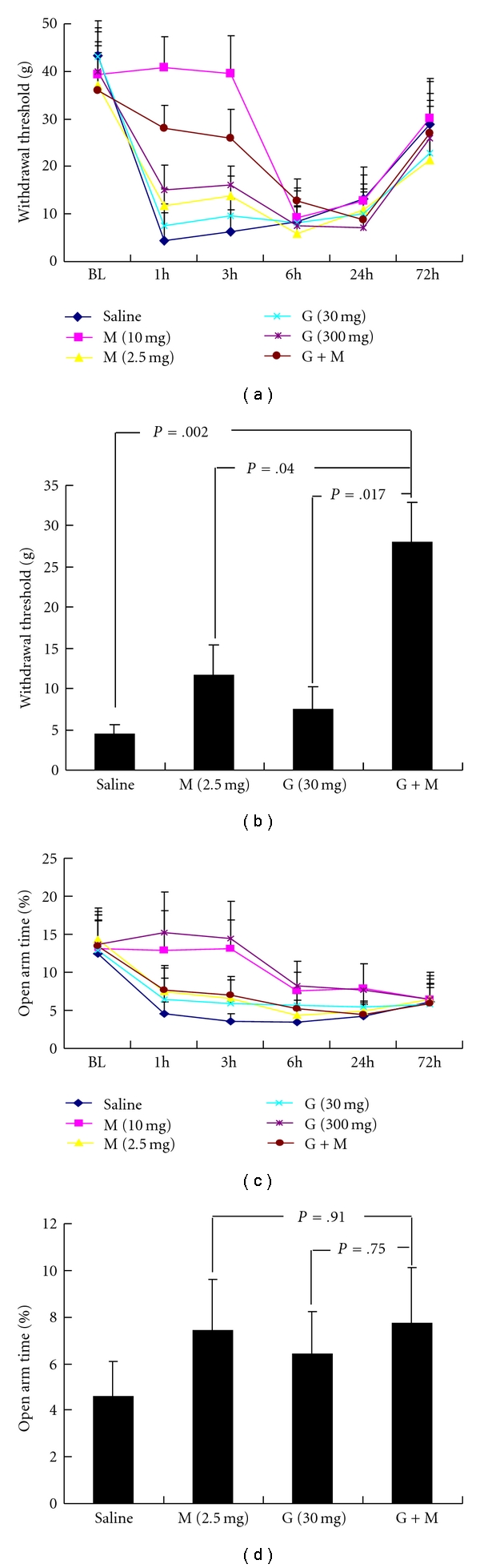
Effect of analgesics on incision-evoked pain hypersensitivity and anxiety-like behavior. Effect of morphine (2.5 mg/kg and 10 mg/kg), gabapentin (30 mg/kg and 300 mg/kg), or the combined morphine (2.5 mg/kg) and gabapentin(30 mg/kg) on the PWT and time spent on open-arm following hind-paw incision. (a) and (c) show the time-course changes in PWT and time spent in open arm in response to surgical and pharmacological treatments. The comparison of PWT and time spent in open-arm between the combined morphine (2.5 mg/kg) and gabapentin(30 mg/kg) and morphine (2.5 mg/kg) or gabapentin (10 mg/kg) at 1 hour after operation are displayed in (b) and (d). G indicates gabapentin; M indicates morphine; G + M indicates the combined treatment of morphine (2.5 mg/kg) and gabapentin (30 mg/kg). *n* = 7 for each group.

 It has been reported that morphine or gabapentin IP administration did not affect pain threshold and time spent the open arm in EPM test in naive rats [18, 20]. Our preliminary study was also consistent with these studies (data not shown). However, 10 mg/kg morphine (IP) treatment could totally reverse the decreased PWT (Figure 4(a)). The antinociceptive effect of morphine sustained 3 hours and disappeared at 6 hours after surgery (Figure 4(a)). The low dose (2.5 mg/kg) of morphine also significantly attenuated the decreased PWT in response to incision (*P* < .05 versus saline treatment, Figure 4(a)). 

 Similarly, pretreatment of gabapentin significantly reversed the decreased PWT induced by incision in a dose-dependent manner. 30 mg/kg of gabapentin pretreatment had only a marginal effect (*P* > .05, Figure 4(a)). However, 300 mg/kg gabapentin reversed PWTs from 4.4 ± 1.0 g to 17.5 ± 2.4 g at 1 hour after incision (*P* < .05 versus basal level or saline treatment, Figure 4(a)). 

In the rats with combined treatment of morphine (2.5 mg/kg) and gabapentin (30 mg/kg), the PWT at 1 hour and 3 hours after surgery are much higher than those in rats treated with 2.5 mg/kg morphine or gabapentin (Figure 4(b)). These data suggest that morphine and gabapentin have a synergistic anti-nociceptive effect. 

Figure 4(c) shows the effect of morphine and gabapentin on the anxiety-like behavior. In the rats with 10 mg/kg morphine treatment, the percentage of time spent in open arm at 1 and 3 hours was similar to the basal level and much higher than those with saline injection (*P* < .01 versus saline treatment, Figure 4(c)). The anxiolytic effect of morphine still existed at 6 hours post operation when the anti-nociceptive effect disappeared (*P* < .01 versus saline treatment, Figure 4(c)). 2.5 mg/kg morphine treatment also had a mild anxiolytic effect. In addition, 300 mg/kg gabapentin reversed the anxiety-like behavior at 1 hour and 3 hours post operation (*P* < .05 versus saline treatment, Figure 4(c)) whereas 30 mg/kg gabapentin had only marginal anxiolytic effect (Figure 4(c)). There was no significant difference in the percentage of time spent in open arm between the group with combined morphine (2.5 mg/kg) and gabapentin (30 mg/kg) treatment and that with morphine (2.5 mg/kg) treatment (Figure 4(d)). These results suggest that morphine and gabapentin have no synergistic anxiolytic effect.

### 4.4. Effect of Morphine and Gabapentin on p-CREB Expression in Amygdala

**Figure 5:**
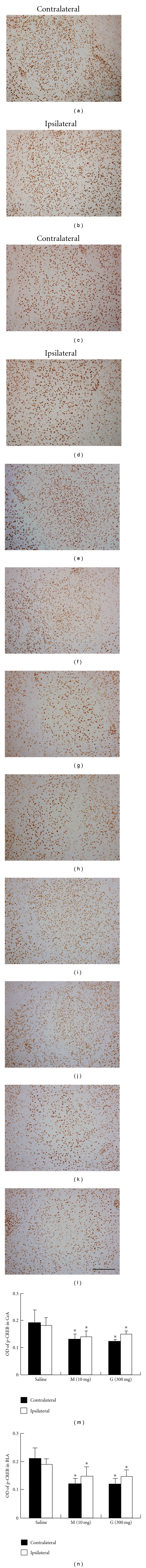
Analgesics inhibited incision-induced amygdala sensitization. (a)–(d) the increased p-CREB immunoreactivity in bilateral CeA (a) and (b) and BLA (c) and (d) in saline-injection at 1 hour following hind-paw incision. (e)–(l) Effect of morphine (e)–(h) and gabapentin (i)–(l) on p-CREB expression in the amygdala at 1 hour after incision. 10 mg/kg morphine inhibited the upregulation of p-CREB immunoreactivity in bilateral CeA (e) and (f) and BLA (g) and (h). 300 mg/kg gabapentin also inhibited the increased p-CREB expression in CeA (i) and (j) and BLA (k) and (l). (m) and (n): Quantitative analysis of the OD of p-CREB immunoreactivity in CeA (m) and BLA (n). Bar, 100 m *P < .05 versus the ipsilateral nucleus in saline-injection control. *n* = 5 for each group.

The present study then investigated the effect of morphine and gabapentin on p-CREB expression in the amygdala. As shown in Figure 5, the increased p-CREB immunoreactivity in the bilateral CeA at 1 hour post operation (Figures 5(a) and 5(b)) was inhibited by 10 mg/kg morphine treatment (Figures 5(e) and 5(f)) or gabapentin treatment (Figures 5(i) and 5(j)). Similarly, the p-CREB expression in the bilateral BLA in the groups treated by morphine (Figures 5(g) and 5(h)) or gabapentin (Figures 5(k) and 5(l)) was significantly less than the control groups at 1 hour after saline injection (Figures 5(c) and 5(d)). These results indicated that morphine and gabapentin could inhibit incision-induced amygdala sensitization as indicated by p-CREB expression.

## 5. Discussion

 The present study sought to investigate the effects of surgery on anxiety-like behavior in an incisional model, an acute state mimicking the clinical postoperative pain. The present study showed that the anxiety-like behavior is also developed after surgical incision in addition to the development of mechanical hypersensitivity, a typical feature of postoperative pain model. 

### 5.1. Development of an Anxiety-Like Behavior in Incisional Pain

Ample clinical evidence shows that chronic pain is associated with negative affective states such as depression and anxiety [2, 3, 6]. Extensive animal studies have also revealed that anxiety-like behavior develops in various chronic pain states [18, 19]. However, understanding the relationship between the anxiety and postoperative pain is quite limited. In the present study, rats with incision spent much less time in the open arm as compared with the sham-operated rats. In addition, open-field test also demonstrated that the time spent in the inner square was reduced significantly at 1 hour post operation. The less time spent in open arm in the incisional rats was not due to the decreased locomotion because the total travel distance was not significantly reduced in response to incision. In addition, the different time courses between the pain and anxiety also support the note that the anxiety behaviour was not due to the pain-evoked reduction in locomotion, as the anxiety behaviours remained at least for 3 days post operation when the pain threshold returned to the normal level. These data showed that the anxiety-like behavior also developed in incisional pain model, as seen in chronic inflammatory pain [5] or neuropathic pain [19]. 

 A recent study showed that anxiety-like behavior was positively correlated with mechanical hypersensitivity in neuropathic pain models in which the lower PWT was associated with less time spent in open-arm in CCI model [14]. In the present study, both of pain hypersensitivity and anxiety-like behavior were rapidly induced implying that the induction of both behaviors was closely related. However, the anxiety-like behavior lasted longer than pain hypersensitivity. The fact that the maintenance of incision-evoked anxiety was not in parallel with the mechanical pain sensation suggests there may be different mechanisms to regulate the maintenance of sensory and affective components of pain.

### 5.2. Incision Induces Central Sensitization in the Amygdala

The amygdala is now recognized as an important player in the emotional-affective dimension of pain [22, 23]. In addition, it has been well known that p-CREB in the brain or spinal cord are closely related with pain processing and mood disorders [24–27]. Thus, the present study used p-CREB as an indicator to investigate the sensitization of amygdala in incisional pain. In response to incision, p-CREB expression was transiently increased in the CeA and BLA suggesting the activation of the amygdala in incisional pain. It has been reported that CeA integrates affective components from the BLA of amygdala with a nociceptive signaling through the spino-parabrachio-amygdaloid pain pathways [22, 23]. In addition, persistent pain was also believed to induce the synaptic plasticity in the CeA, and deactivating it would decrease the nocifensive and affective pain [25, 26]. The activation of amygdala after incision suggests that there may be a similar central sensitization between acute pain and chronic pain, and the deactivation of the amygdala might be a potential intervention for the treatment of postoperative pain-related anxiety.

### 5.3. Effect of Morphine and Gabapentin on Anxiety-Like Behavior

Recent study showed that the analgesic drugs, morphine, attenuated the anxiety-like behavior in neuropathic pain [14]. In the present study, 2.5 mg/kg morphine attenuated the anxiety-like behavior, whereas 10 mg/kg could entirely reverse it implying that its dose-dependent anxiolytic effect. However, in spite of its total antinociceptive and anxiolytic effect, the dosage (10 mg/kg) used in the present study may have severe side effects such as physical dependence or tolerance [27, 28]. Thus, it may be impractical to use such high dosage clinically and other drugs may be also considered to treat the operation-induced anxiety and pain sensation. 

 Gabapentin, an anticonvulsant drug with anti-nociceptive property, has been used in the postoperative pain [29, 30]. Experimental studies reported that 30 mg/kg gabapentin exerted the anxiolytic effect as well as the antinociceptive effect in neuropathic pain [14]. In the present study, 30 mg/kg gabapentin obviously reversed the incision-evoked pain hypersensitivity but had only marginal anxiolytic effect. These results indicate that the dose of 30 mg/kg that is widely used in the animal model mainly exerts the antinociceptive effect. In this regard, gabapentin has been shown to suppress the release of excitatory amino acids in the spinal cord induced by incision [31]. On the other hand, the dose of 300 mg/kg gabapentin not only greatly alleviated incision-evoked pain hypersensitivity, but also entirely reversed the incision-induced anxiety. This suggests that the higher dose of gabapentin has its own anxiolytic effect through directly acting on the brain areas related to the affective dimension. Supporting this hypothesis, 300 mg/kg of gabapentin also inhibited the activation of p-CREB in the amygdala. However, the dose of 300 mg/kg was also very high and may not be applicable to clinics. 

In clinical trials, pre-operative gabapentin administration could attenuate the postoperative pain intensity and reduce the consumption of opioids [29, 30]. In agreement with this, the combined morphine and gabapentin treatment in the present study synergistically reversed the pain hypersensitivity incision. The synergistic antinociceptive effect of morphine and gabapentin may be due to their separate action on the peripheral and central nervous system [32]. However, the EPM test revealed that, unlike their synergistic antinociceptive effect, morphine and gabapentin had no synergistic anxiolytic effect because there was no significant difference of time spent in open-arm between the group with combined gabapentin and morphine treatment and the group with single morphine treatment. These data also indicate that the development of anxiety was not closely correlated with pain hypersensitivity corresponding to surgical incision and further support the hypothesis that distinct mechanism regulates the maintenance of pain sensation and pain-related anxiety. In this situation, after surgical trauma, the nociceptive signaling was likely transported through the spinothalamic and corticolimbic pathways that finally connect to the brain circuits including amygdala, which in turn contributed to the anxiety-like behavior. However, once the central sensitization has occurred, the anxiety may be independent of the sensory component and exists without the involvement of sensory input.

## 6. Conclusion

The present study showed that surgical incision could induce the pain-related anxiety behavior as accompanied with the expression of p-CREB in the amygdala, the brain area related to the mood disorder and pain processing. The analgesic drugs, morphine and gabapentin, could attenuate the incision-induced anxiety and the activation of p-CREB in the amygdala. Thus, administration of gabapentin and/or morphine to treat surgery-induced affective disturbance may be useful in postoperative pain management.
